# Super-Resolution Imaging of Higher-Order Chromatin Structures at Different Epigenomic States in Single Mammalian Cells

**DOI:** 10.1016/j.celrep.2018.06.085

**Published:** 2018-07-24

**Authors:** Jianquan Xu, Hongqiang Ma, Jingyi Jin, Shikhar Uttam, Rao Fu, Yi Huang, Yang Liu

**Affiliations:** 1 Biomedical Optical Imaging Laboratory, Departments of Medicine and Bioengineering, University of Pittsburgh, Pittsburgh, PA 15213, USA; 2 School of Medicine, Tsinghua University, No.1 Tsinghua Yuan, Haidian District, Beijing 100084, China; 3 Department of Computational and Systems Biology, University of Pittsburgh, Pittsburgh, PA 15213, USA; 4 College of Chemical Engineering, Northeast Electric Power University, Jilin City, Jilin Province 132012, China; 5 Magee-Women’s Research Institute, University of Pittsburgh Cancer Institute, 204 Craft Avenue, Pittsburgh, PA 15213, USA; 6 Lead Contact

## Abstract

Histone modifications influence higher-order chromatin structures at individual epigenomic states and chromatin environments to regulate gene expression. However, genome-wide higher-order chromatin structures shaped by different histone modifications remain poorly characterized. With stochastic optical reconstruction microscopy (STORM), we characterized the higher-order chromatin structures at their epigenomic states, categorized into three major types in interphase: histone acetylation marks form spatially segregated nanoclusters, active histone methylation marks form spatially dispersed larger nanodomains, and repressive histone methylation marks form condensed large aggregates. These distinct structural characteristics are also observed in mitotic chromosomes. Furthermore, active histone marks coincide with less compact chromatin and exhibit a higher degree of co-localization with other active marks and RNA polymerase II (RNAP II), while repressive marks coincide with densely packed chromatin and spatially distant from repressive marks and active RNAP II. Taken together, super-resolution imaging reveals three distinct chromatin structures at various epigenomic states, which may be spatially coordinated to impact transcription.

## In Brief

Using STORM-based super-resolution microscopy, Xu et al. discover that genome-wide higher-order chromatin structures at various epigenomic states form three types of distinct structures: segregated nanoclusters, dispersed nanodomains, and compact large aggregates. Their spatial relationship with each other and RNA polymerase II suggests spatial coordination that impacts transcription.

## Graphical Abstract

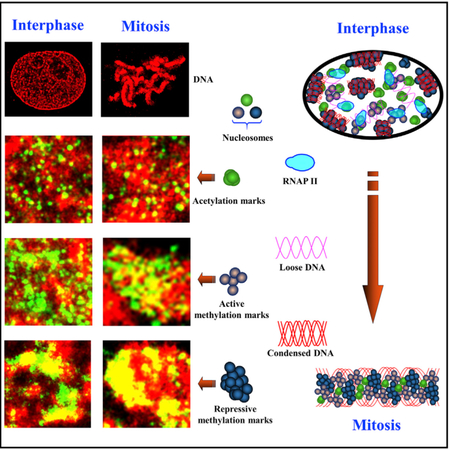

## INTRODUCTION

Eukaryotic cells package genomic DNA up to 2 m long into a nucleus with a diameter of several microns through a hierarchical scheme of compaction into DNA-protein assemblies. The first level is nucleosome, consisting of 147 bp of DNA wrapped around an octamer of four core histone (H2A, H2B, H3, and H4) proteins. This basic repeating unit of nucleosomes is then organized into ~10-nm “beads-on-string” chromatin fiber, which is further compacted into a higher-order chromatin structure, to fit into the micron-sized nucleus. Chromatin organization is regulated by a large number of chemical modifications, particularly on the N-terminal tails of histone core proteins, such as acetylation and methylation. Histone modifications regulate the packaging of nucleosomes into a higher-order chromatin structure to influence the accessibility of genomic DNA to the transcription machinery proteins. Subsequently, chromatin compaction at different epigenomic states controls their gene expression (Strahl and Allis, 2000; Turner, 2000) and imposes a significant effect on many cellular processes, such as DNA replication, cell division, DNA damage, and DNA repair.

How different histone modifications shape the higher-order chromatin structure at each epigenomic state remains an important question (Cortini et al., 2016). Due to the limited resolution of conventional light microscopy, our current understanding of higher-order chromatin structures defined by different histone modifications is indirectly inferred from *in vitro* biochemical assays such as chromatin immunoprecipitation (ChIP) (Patel and Wang, 2013; Zhou et al., 2011) and chromatin conformation capture (Dekker et al., 2013). These assays often rely on the analysis of fragmented DNA from pooled cell population and lose the information at a single-cell level. Recent advance in super-resolution fluorescence microscopy now enables the imaging of chromatin structures below the diffraction-limited resolution in both fixed and live cells. Structured illumination microscopy (SIM) (Gustafsson, 2000) was used to reveal nuclear topography and functional chromatin domains (Cremer et al., 2017; Markaki et al., 2010). Photoactivated localization microscopy [PALM] (Betzig et al., 2006) was used to visualize the higher-order chromatin structures and their dynamics in live mammalian cells (Nozaki et al., 2017). Stimulated emission depletion (STED) microscopy (Hell and Wichmann, 1994) was used to measure chromatin features in mammalian cells (Mitchell-Jordan et al., 2012; Monte et al., 2016). Localization-based super-resolution microscopy, such as (direct) stochastic optical reconstruction microscopy (STORM) (Rust et al., 2006; van de Linde et al., 2011), offers one of the best spatial resolutions to directly visualize the previously invisible higher-order chromatin structure down to an optical resolution of 20–30 nm *in vivo* in a singlecell nucleus. Super-resolution imaging revealed that chromatin structures *in vivo* consist of heterogeneous groups of nucleo-some clusters (Prakash et al., 2015; Ricci et al., 2015), as well as distinct chromatin packaging for different epigenomic states at specific gene loci (Boettiger et al., 2016). However, the *in situ* genome-wide higher-order chromatin structures formed by different histone modifications remain elusive.

In this study, we focus on a comprehensive *in situ* characterization of genome-wide higher-order chromatin structures defined by histone acetylation and methylation marks and their spatial proximity that collectively form the chromatin environment in single mammalian cell nuclei via STORM. We selected a set of 10 histone marks, including lysine acetylation involved in active transcription and lysine methylation involved in repressive and active transcription. Our super-resolution imaging and quantitative analysis reveal three major structural characteristics of higher-order chromatin: histone acetylation forms spatially segregated nucleosome nanoclusters, active histone methylation forms spatially dispersed nucleosome nanodomains, and repressive histone methylation forms highly condensed large aggregates. Two-color STORM imaging shows that the transcriptionally active histone mark coincides with “open” chromatin and that the transcriptionally repressive histone mark coincides with highly condensed chromatin. Further examination of their spatial proximity show that repressive and active histone marks are mostly spatially exclusive, while considerable co-localization can be observed among active histone marks. Taken together, super-resolution imaging helps reveal how histone acetylation and methylation form the higher-order chromatin structures at a scale ranging from tens of nanometers to a few microns at a level of the single mammalian cell nucleus.

## RESULTS

### Three Distinct Structural Characteristics of Higher-Order Chromatin Structure Formed by Histone Marks in the Interphase Nuclei

**Figure 1.**
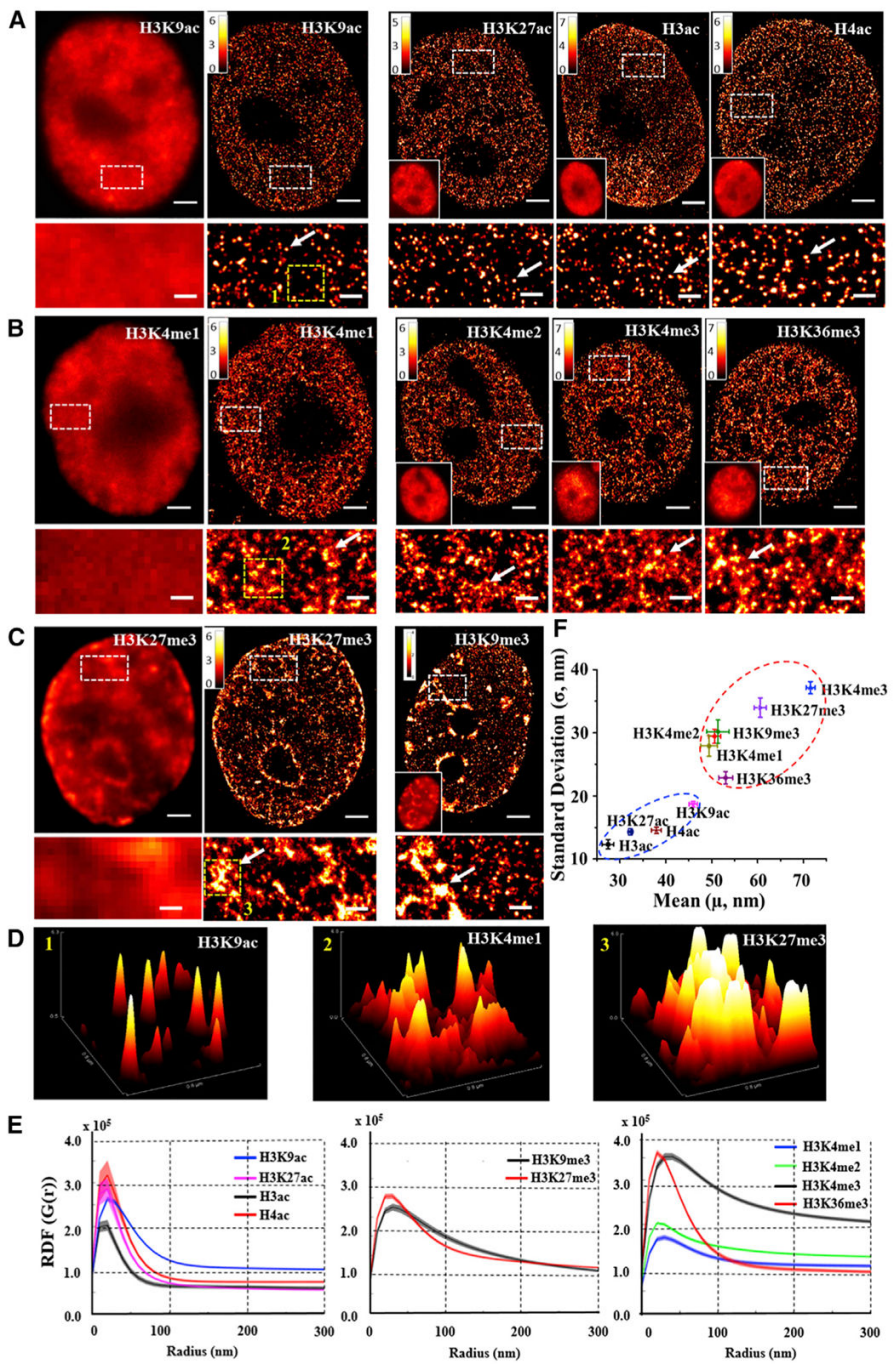
The STORM Images and Quantitative Characterization of Different Histone Marks (A–C) The representative wide-filed and STORM images of three groups of histone modifications: (A) histone acetylation marks, (B) repressive marks, and (C) active histone methylation marks. The rest of the wide-filed images in each group are shown as inset figures. White arrows emphasize the featured structure of different histone marks. Scale bars, 2 μm and 500 nm in the original and magnified images, respectively. (D) Overall distribution pattern of the three representative histone marks (H3K9ac, H3K4me1, and H3K27me3) shown as the surface plot from the selected region in the yellow boxes in (A)–(C). (E) The average radial distribution for each histone mark categorized into the above three groups. The solid curve indicates the average value from all nuclei (~20–40 nuclei), and the shaded area indicates the SE. (F) The scatterplot of the mean size versus SD (averaged over ~20–40 cells) for 10 histone marks in (A)–(C). Error bars indicate SE. See also Figure S1.

We first visualized the genome-wide higher-order chromatin structures defined by a set of 10 histone modifications in the mammalian cell nuclei. Figures 1A–1C show representative conventional wide-field and super-resolution images from 10 histone marks, including transcriptionally active histone acetylation marks (H3K9ac, H3K27ac, H3ac, and H4ac), transcriptionally active histone methylation marks (H3K4me1, H3K4me2, H3K4me3, and H3K36me3), and transcriptionally repressive histone methylation marks (H3K27me3 and H3K9me3) (additional images of each histone mark are shown in Figure S1). The wide-field images reveal little difference in their higher-order chromatin structure between active histone acetylation and active histone methylation marks, as both exhibit relatively homogeneous distribution within the cell nuclei (Figures 1A and 1B). However, their super-resolution images reveal distinct structural features: histone acetylation marks form spatially segregated and discrete nucleosome nanoclusters with a narrow size distribution, while histone methylation marks form highly heterogeneous and spatially dispersed nucleosome nanodomains. The spatially segregated nanoclusters from histone acetylation marks are present not only for histone modification on a specific lysine residue (e.g., H3K9ac and H3K27ac) but also for pan-acetylation that targets multiple acetylated lysine residues (e.g., pan-H3ac and pan-H4ac).

On the other hand, the repressive marks (H3K27me3 and H3K9me3) shown in the wide-field image of Figure 1C exhibit condensed aggregates in the nucleus. The super-resolution images clearly show the presence of highly condensed mega-sized (at a scale of a few hundred nanometers) and even super-sized (micron-sized) clumps enriched at the periphery of the nucleus, nucleolus, and nucleoplasm, consistent with our conventional view of heterochromatin observed under electron microscopy (Heitz, 1928; van Steensel, 2011). In contrast to active histone marks, such a prominent presence of highly condensed large aggregates enriched at the nuclear periphery is highly characteristic of repressive histone methylation marks. Figure 1D shows the surface plot of the overall distribution pattern of three representative histone marks (H3K9ac, H3K4me1, and H3K27me3) from the selected regions of the super-resolution images in Figures 1A–1C. It clearly shows the three distinct characteristic features of the higher-order chromatin structure: the spatially well-separated “peaks” formed by the histone acetylation mark, the spatially dispersed pattern formed by the active histone methylation mark, and the highly condensed large clumps formed by the repressive histone methylation mark.

We further quantified these structural characteristics formed by each histone mark. As shown in Figures 1E and 1F, we performed spatial clustering by two different methods – radial distribution function (RDF) (Figure 1E; Caetano et al., 2015) and Gaussian clustering (Figure 1F; Ma et al., 2017; Ricci et al., 2015). RDF (G(r)) is also known as a pair-correlation function and quantifies the probability of finding a molecule with respect to its neighboring molecules as a function of radial distance r. The width of the RDF indicates the correlation length, and the height of the RDF indicates the relative degree of clustering. For the randomly distributed molecules, G(r) will remain constant at all radial distances. Figure 1E shows that histone acetylation marks exhibit narrow sharp peaks at a short length scale less than 50 nm, suggesting the presence of highly clustered small nanosized structures. On the other hand, the RDFs of histone methylation marks exhibit a much wider distribution, suggesting the presence of larger aggregates and longer correlation length. We further quantified the size of the nanoclusters and nano-domains formed by different histone marks, and Figure 1F shows the scatterplot of mean size versus SD. Histone acetylation marks occupy the lower left corner, with the mean size smaller than 50 nm and an SD of less than 20 nm, but histone methylation marks occupy the upper right corner, with the mean size of larger than 50 nm and a wider SD of 30–40 nm. The results from the quantitative analysis are consistent with our qualitative visualization of the superresolution images of histone acetylation and methylation marks (Figures 1A–1C).

To further confirm the structural distinction between histone acetylation and histone methylation marks, we induced histone hyper-acetylation by treating the cells with histone deacetylase inhibitor (suberoylanilide hydroxamic acid [SAHA]). As shown in Figure S2, we confirmed that the characteristic spatially segregated nanoclusters from the histone acetylation mark are stil maintained, despite the increased size of nanoclusters due to the increased acetylation. In addition, to rule out the possibility of potential image artifacts due to different labeling density, we deliberately varied the labeling density of histone acetylation (H4ac) and methylation (H3K4me3) marks. As shown in Figure S2, although the number of the localized spots at each cluster increases with increasing labeling density, we can still observe the characteristic structural distinction between the spatially segregated nanoclusters formed by the histone acetylation mark and the spatially diffuse nanodomains formed by the active histone methylation mark. This structural distinction is also reflected in the corresponding RDF (Figure S2), which shows the narrow and sharp distribution with small correlation length formed by the histone acetylation mark and the wider distribution with longer correlation length formed by the histone methylation mark.

To rule out that the characteristic structural features formed by different histone marks are not cell-type specific, we imaged different cell lines (mouse embryonic fibroblast [MEF] and U2OS cells). Figure S2 shows the super-resolution images and the corresponding RDF distribution. The three distinct characteristic features are still observed in these cell lines: the histone acetylation mark (H4ac) shows small spatially segregated nanoclusters with a narrow RDF, the active histone methylation mark (H3K4me3) forms dispersed larger nano-domains with a broader RDF, and the repressive histone methylation mark (H3K27me3) forms highly condensed large clumps.

### Association between Chromatin Compaction and Histone Marks

**Figure 2.**
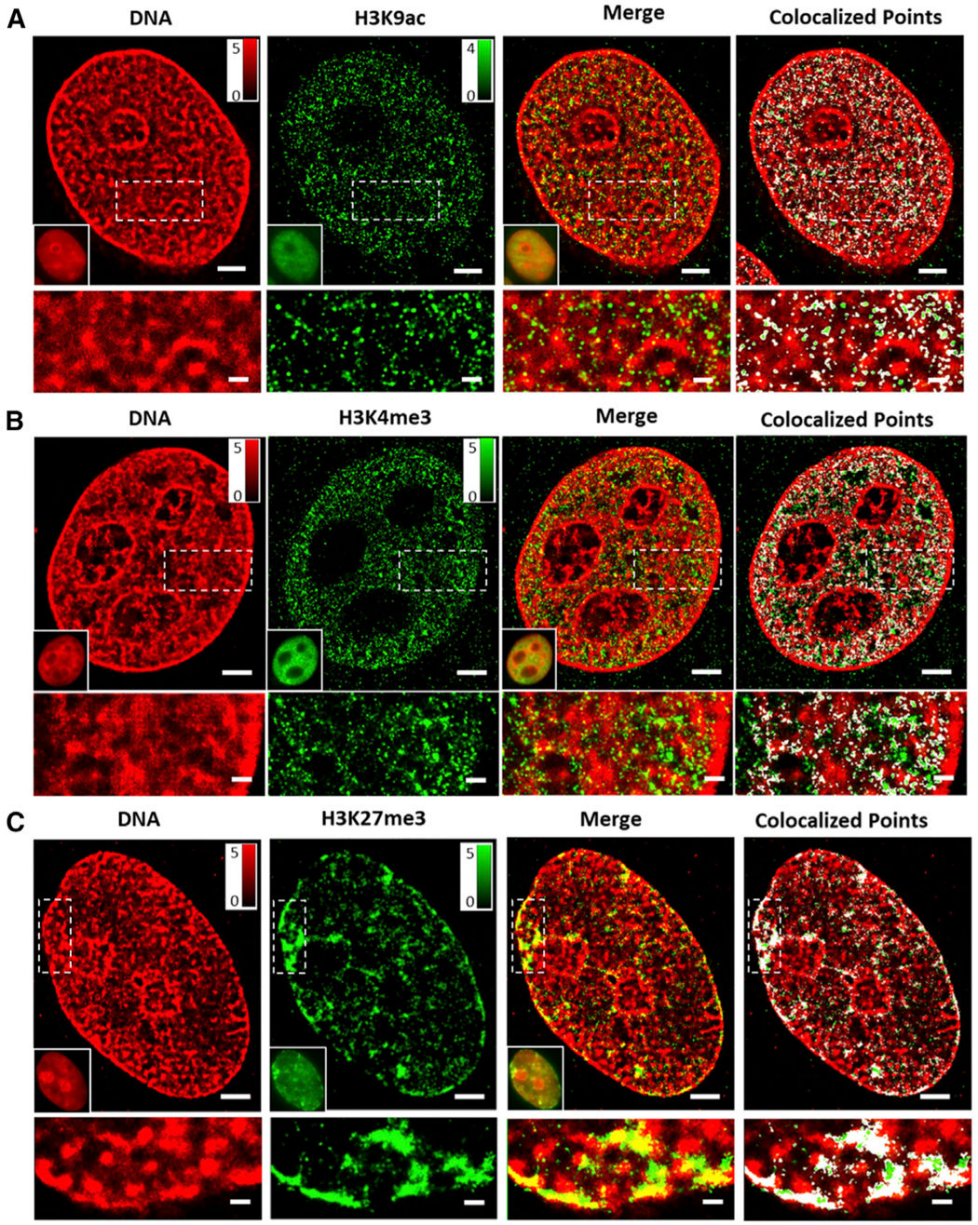
Two-Color STORM Images of Different Histone Marks and DNA in the Interphase Nuclei (A–C) The representative two-color STORM images showing the spatial relationship between DNA and different histone modifications, ncluding (A) active histone acetylation H3K9ac, (B) active histone methylation H3K4me3, and (C) repressive histone methylation H3K27me3. Green indicates histone marks (labeled by Cy3B), and red indicates DNA (labeled by Alexa Fluor 647). White spots indicate the colocalized points. Scale bars, 2 μm and 500 nm in the original and magnified images, respectively. See also Figure S3.

Next, we visualized the spatial relationship between histone marks and DNA via two-color STORM imaging. Figure 2 shows the representative super-resolution images of transcriptionally active or repressive histone marks (green, labeled with Cy3B) and DNA (red, labeled with Alexa Fluor 647), as well as their merged images and co-localized points (marked in white) (additional images can be found in Figure S3). These images reveal that DNA is highly compact and forms compartmentalized regions in the cell nuclei, where the active histone marks of H3K9ac or H3K4me3 are more abundant at the less compact regions of DNA (Figures 2A and 2B), and the repressive histone mark of H3K27me3 mostly coincides with the condensed regions of DNA (Figure 2C). This direct visual evidence shows the relationship between histone marks and chromatin compaction: repressive histone marks are associated with highly condensed chromatin structures, and active histone marks are associated with more “open” or less compact chromatin structures. To quantify the chromatin structure, three regions are classified based on k-means clustering: open, intermediate, and condensed, as shown in Figures S4A–S4C. The number of localized spots in the condensed chromatin regions is about 6 times that in the open chromatin region. The H3K4me3 clusters exhibit the highest density in the open chromatin region, while H3K27me3 clusters exhibit the highest density in the condensed chromatin regions (Figures S4D and S4E). Therefore, both qualitative visualization and quantitative analysis confirm that the structural characteristics from different histone marks, as observed earlier, are, indeed, representative of their associated higher-order open or condensed chromatin structures.

### Distinct Structural Characteristics of Histone Marks at the Mitotic Phase

**Figure 3.**
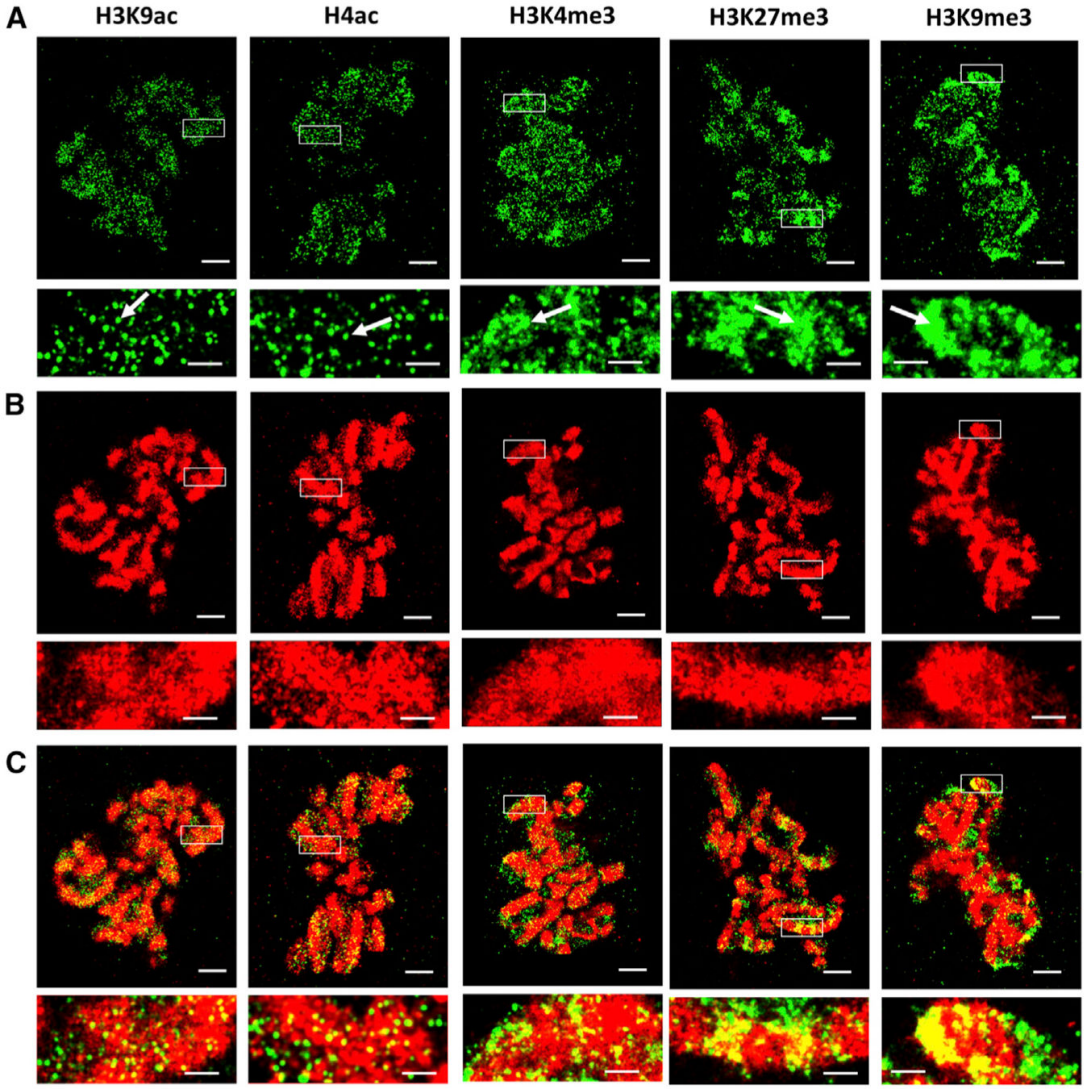
The Representative Two-Color STORM Images of Different Histone Modification Marks and DNA in the Mitotic Phase Green channel (A) indicates histone marks (labeled by Cy3B), and red channel (B) indicates DNA (labeled by Alexa Fluor 647). (C) Merged channel. Arrows indicate the presence of the preserved structure characteristic of each histone mark. Scale bars, 2 μm and 500 nm in the original and magnified images, respectively.

The structural characteristics observed, as described earlier, under super-resolution microscopy are from the interphase nuclei where chromatin is generally “loosely” distributed. When cells undergo mitosis, chromatin structure reorganizes and condenses into micron-sized chromosomes visible under a conventional light microscope. We next explored the higher-order chromatin structure formed by each histone mark (green, labeled with Cy3B) and associated DNA (red, labeled with Alexa Fluor 647) during mitosis, as shown in the super-resolution images in Figure 3. The spatial distribution of histone marks generally coincide with DNA in the mitotic phase. Interestingly, despite the highly compacted chromosomes, the characteristic structural features formed by each histone mark in the interphase nuclei can also be seen in the chromosomes during mitosis, such as the discrete nanoclusters in H4ac and H3K9ac, the spatially dispersed nucleosome nanodomains in H3K4me3, and the highly condensed large clumps in H3K27me3 and H3K9me3, as indicated by the white arrows in Figure 3A. This result suggests that this highly conserved unit of nucleosome clusters in the mitosis may play a role in maintaining the assembly of higher-order chromatin structures through cell division.

### Spatial Proximity between Different Histone Marks

**Figure 4.**
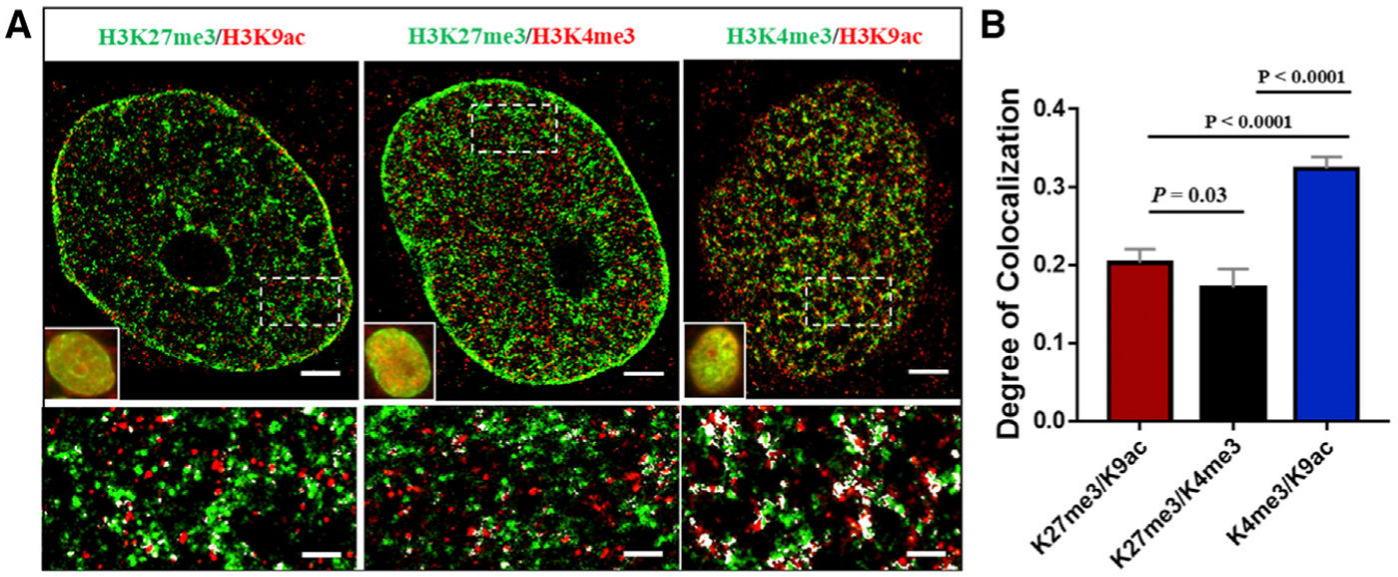
Two-Color STORM Images of Different Histone Modification Marks (A) Representative STORM image showing spatial relationship among different histone modifications, categorized into three pairs: active versus repressive histone marks (H3K27me3 versus H3K9ac), bivalent histone marks (H3K27me3 versus H3K4me3), and two active histone marks (H3K4me3 versus H3K9ac). White spots indicate the co-localized spots. Scale bars, 2 μm and 500 nm in the original and magnified images, respectively. (B) Statistical analysis of the degree of colocalization (DoC) between each pair of histone modifications. Data are represented as means ± SEM, and the p values were determined using the Mann-Whitney test.

To further characterize the chromatin environment defined by these higher-order chromatin structures at different epigenomic states, we examined the spatial relationship between different histone marks by mapping their spatial proximity for three pairs: repressive versus active histone marks, bivalent histone marks, and two active histone marks, via two-color STORM imaging. As shown in Figure 4, the repressive histone mark H3K27me3 and the active histone mark H3K9ac mostly occupy distinct space, with a degree of co-localization (DoC) of ~0.20 (a DoC of 1 indicates 100% co-localization). The bivalent H3K27me3 and H3K4me3 domains (Barski et al., 2007; Bernstein et al., 2006) exhibit a similar DoC with only a slight decrease (~0.17, p = 0.03), suggesting similar spatial proximity to the repressive and active marks. For the two active histone marks of H3K4me3 and H3K9ac that have previously shown to have coordinated activity (Ghare et al., 2014; Ha et al., 2011), we observed a significantly larger DoC (~0.33, p <0.0001), suggesting that the significantly increased spatial proximity between two active histone marks on the same cell may facilitate more crosstalk. To rule out the potential projection effect on the measured co-localization from the 2D images (i.e., co-localized molecules in the 2D image may be spatially separated in the axial direction), we performed astigmatism-based two-color three-dimensional (3D)-STORM imaging (Huang et al., 2008) and calculated the DoC from the 2D images projected from 300 nm, 160 nm, and 80 nm (the approximate axial resolution of 3D-STORM), respectively. As shown in the results from the 3D-STORM imaging (Figure S5A), most co-localized clusters in the 2D image also appear to be co-localized along the axial direction. Further, Figure S5B suggests that the DoC derived from 2D images of a ~300 nm-thick section is highly correlated with the results from the 3D images.

### Spatial Proximity between Active RNAP II and Histone Marks

**Figure 5.**
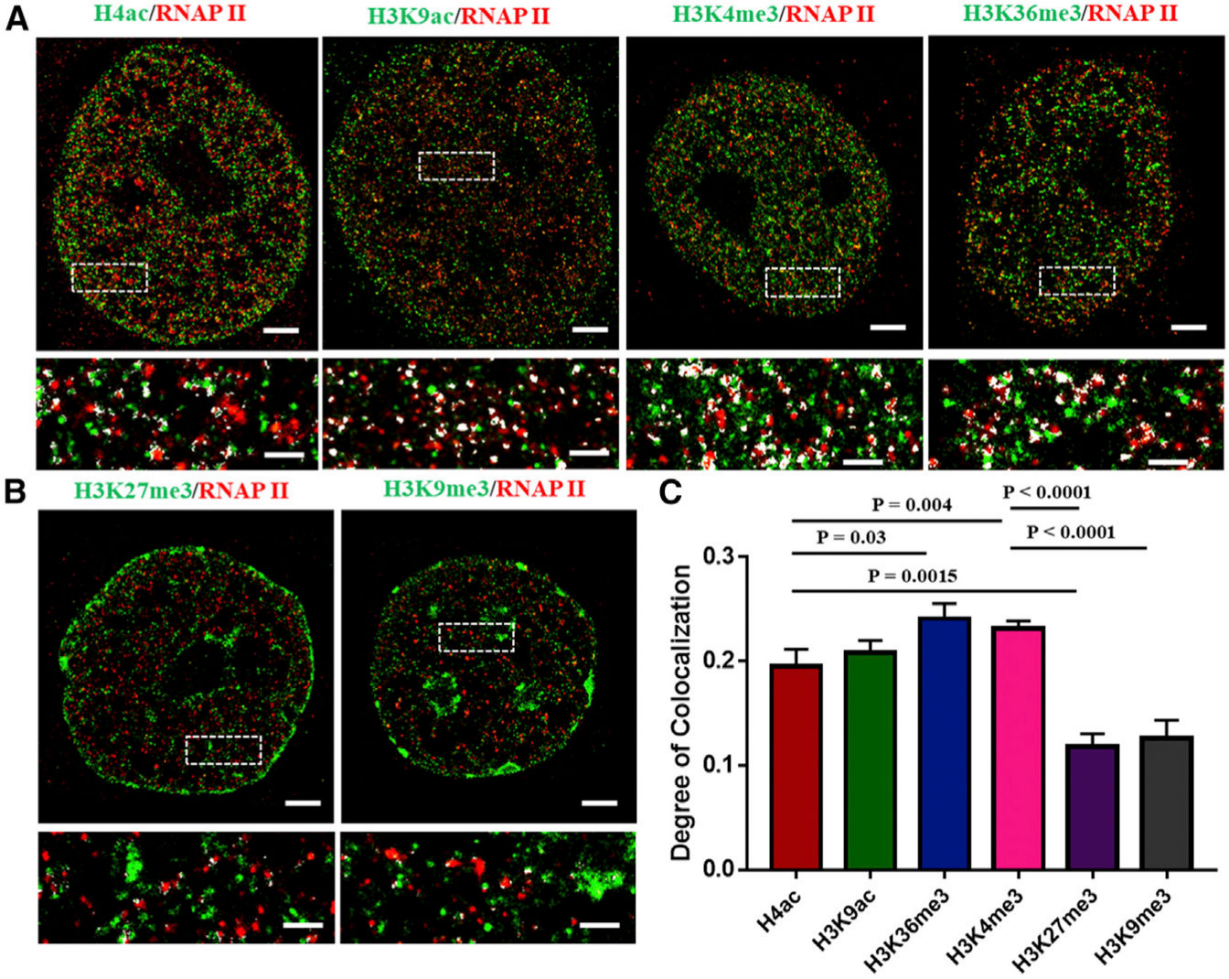
Representative STORM Images of the Spatial Relationship between Histone Marks and Active RNAP II, or Phosphorylated RNAP II (A) Active histone marks (H4ac, H3K9ac,H3K4me3, and H3K36me3) versus active RNAP II. (B) Repressive histone marks (H3K27me3, H3K9me3) versus RNAP II. White spots show the co-localized spots. (C) Statistical analysis of the degree of co-localization (DoC) between different histone marks and RNAP II. Data are represented as means ± SEM, and the p values were determined using the Mann-Whitney test. Scale bars, 2 μm and 500 nm in the original and magnified images, respectively.

One of the major functional consequences of histone modification is the regulation of transcription activities. In eukaryotes, transcription occurs in discrete RNA polymerase II (RNAP II) clustered foci termed “transcription factories” (Chen et al., 2016; Ricci et al., 2015; Zhao et al., 2014), where the largest subunit (RPB1) of RNAP II contains a repetitive carboxy-terminal domain (CTD). Ser-5 of the CTD becomes phosphorylated during transcription and, therefore, can be used as a marker for active transcription (Phatnani and Greenleaf, 2006; Srivastava and Ahn, 2015; Sutherland and Bickmore, 2009). Here, we use two-color STORM to visualize the active form of phosphorylated RNAP II and histone marks to evaluate whether the regions with transcriptionally active histone marks recruit more active RNAP II. Figure 5 shows the spatial proximity between phosphorylated RNAP II and different histone marks, including four active marks (H4ac, H3K9ac, H3K4me3, and H3K36me3) and two repressive marks (H3K9me3 and H3K27m3). Overall, the DoC is significantly higher between active RNAP II and active marks than that between active RNAP II and repressive marks (p < 0.0001). The highest DoC is seen in H3K4me3 and H3K36me3 associated with transcription initiation and elongation, respectively (Guenther et al., 2007). Interestingly, the two-color STORM images also show that the active RNAP II clusters are located side by side with histone marks, in good agreement with previous published results (Markaki et al., 2010; Nozaki et al., 2017) and the proposed model of “transcription factories” (Feuerborn and Cook, 2015). These results suggest that the active transcription machinery is in close proximity to the active histone marks but that it is spatially distant from the repressive histone marks.

## DISCUSSION

We demonstrate the potential of localization-based superresolution microscopy based on STORM, to directly visualize the genome-wide higher-order chromatin structure at their distinct epigenomic states down to a resolution of ~20–30 nm in the nucleus of single mammalian cells. The super-resolution images of a set of histone marks reveal three distinct structural characteristics in higher-order chromatin structure: histone acetylation marks form spatially segregated nanoclusters, active histone methylation marks form spatially dispersed and heterogeneous nanodomains, and repressive histone methylation marks form highly condensed large clumps. The distinct structural characteristics may be a reflection of the different mechanisms of histone acetylation and methylation to regulate chromatin structures (Cortini et al., 2016). It has been well established that histone acetylation directly affects chromatin structures by removing the positive charge on the histone tails and reducing the affinity of tails to adjacent nucleosomes (Shogren-Knaak et al., 2006), thus preventing the formation of densely packed large nucleosome domains. The observed spatially segregated nanoclusters formed by histone acetylation marks may be the structural consequence of the reduced interactions between adjacent nucleosomes. Such spatial segregation may help in maintaining a more “open” chromatin structure; therefore, histone acetylation marks are generally associated with active transcription. In contrast, it has also been shown that histone methylation indirectly affects chromatin structure by reserving the charge of the histone proteins that introduces significant steric hindrance and serving as binding sites to recruit additional architectural proteins (Pasini et al., 2010; Patel and Wang, 2013). The structural consequences of such an indirect effect on chromatin may result in more complex, highly heterogeneous, and spatially dispersed structural characteristics of histone methylation observed in the super-resolution images.

We further confirmed that the active histone marks, indeed, spatially coincide with more “open” or less compact chromatin structure and that the repressive histone marks spatially coincide with densely packed chromatin structure. This result is in good agreement with the previous studies of *Drosophila* chromosomes (Żurek-Biesiada et al., 2016) that active RNAP II clusters were present in the low-density DNA regions of polytene chromosomes. Another interesting finding revealed by superresolution imaging is that the distinct structural features formed by histone marks (e.g., spatially segregated nanoclusters formed by histone acetylation and spatially dispersed nanodomains formed by active histone methylation marks) can even be observed within the highly condensed chromosome in the mitotic phase. This observation is also consistent with previously published results. Nozaki et al. used live-cell imaging based on PALM and reported that the compact chromatin domains that exist in the interphase nuclei also exist during mitosis (Nozaki et al., 2017). Ou et al. used chromatin electron microscopy (ChromEM) tomography and found that the overall primary structure of chromatin packed at different densities does not change in the mitotic chromosomes (Ou et al., 2017). The preserved features of the basic structural unit of nanoclusters or nanodomains formed by individual histone modifications during mitotic phase may facilitate the inheritance of epigenomic modifications through cell division (Budhavarapu et al., 2013; Moazed, 2011).

We further characterized the chromatin environment defined by the spatial proximity between different histone marks and their association with active transcription machinery RNAP II. The little co-localization between active and repressive histone marks suggests the establishment of distinct domains between transcriptionally active and repressive histone marks, which also agrees with the previous observation on the little overlap between repressed and active chromatin domains (Boettiger et al., 2016). The significantly higher co-localization between active marks suggests that their closer proximity may facilitate their interaction or cross-talk (Ghare et al., 2014; Ha et al., 2011). Similarly, the higher DoC between the active transcription factory enriched by phosphorylated RNAP II and active histone marks (H3K9ac, H4ac, H3K36me3, and H3K4me3) suggests that their spatial proximity may facilitate their interaction, consistent with those results inferred by conventional biochemical assays (Crump et al., 2011; Wang et al., 2009).

**Figure 6.**
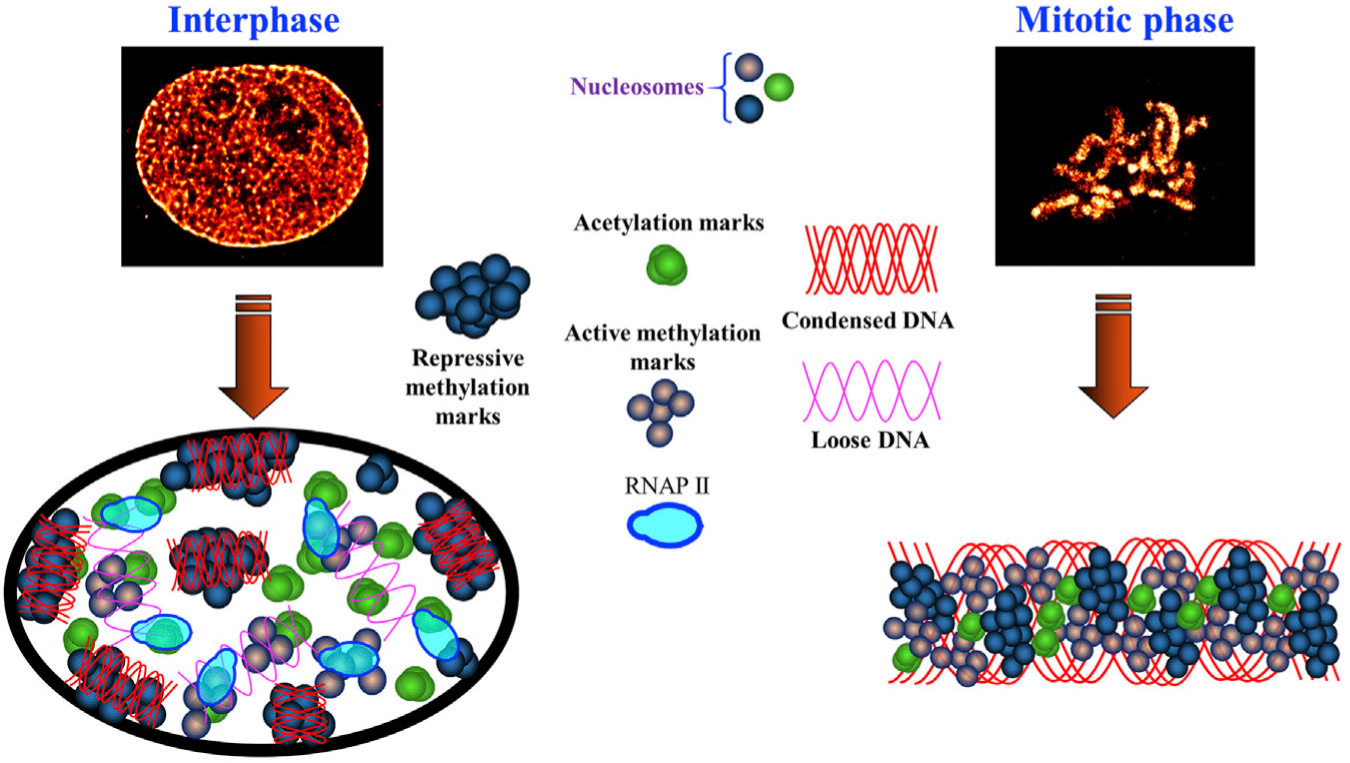
Model to Depict the Spatial Organization of Chromatin Model to illustrate the spatial organization of the chromatin environment at interphase and mitotic phase composed of three distinct groups of structural characteristics from active histone acetylation, active histone methylation, and repressive histone methylation, as well as their spatial relationship with active transcription machinery.

Our results suggest a model for higher-order chromatin structure at their distinct epigenomic states and their associated chromatin environment, as illustrated in Figure 6. The higher-order chromatin structure at different epigenomic states is formed by the packaged DNA and various histone proteins and can be mainly categorized into three distinct structural characteristics: (1) spatially segregated nanoclusters formed by active histone acetylation marks, (2) spatially dispersed and highly heterogeneous nanodomains formed by active histone methylation marks, and (3) highly condensed large clumps formed by repressive histone methylation marks. The active and repressive chromatin domains are often spatially exclusive, while active marks are in closer proximity with each other. The open chromatin formed by active histone marks allows the closer access to active transcription machinery. Furthermore, these characteristic structural features formed by individual histone marks were maintained at the mitotic phase. This model is also in good agreement with the model of nanoscale structure of the pachytene chromosomes (Prakash et al., 2015) and the chromatin domain structure model (Nozaki et al., 2017).

We have also taken several precautions in the interpretation of our super-resolution imaging results. As with the findings of other biochemical analyses of histone modifications, our results rely on antibody specificity. All antibodies used in this study were well characterized by western blotting and immunofluorescence staining (information is shown in Table S1). To rule out the potential artifact due to some non-specific binding of certain antibodies, we validated the presence of similar structural characteristics formed by a specific histone mark labeled by the independently derived antibodies from different manufacturers (H3K4me2, EMD Millipore, 07-030; H3K4me2, Abcam, ab7766; H3K4me3, EMD Millipore 04-473; and H3K4me3, abcam, ab8580). For two-color super-resolution imaging where antibodies from different host species are required, we validated that the super-resolution images of histone marks labeled by antibodies from different hosts present similar structural characteristics (mouse anti-H3K9ac, Abcam, ab12179; rabbit anti-H3K9ac, EMD Millipore, 07-352; mouse anti-RNAP II, Abcam, ab5408; rabbit anti-RNAP II, Abcam, ab5131), as shown in Figure S6.

### Conclusions

In conclusion, we demonstrate the potential of super-resolution localization microscopy to directly visualize the genome-wide higher-order chromatin structure at their epigenomic states in the interphase and mitotic mammalian cell nuclei. Our results reveal the previously unseen distinct characteristics of higher-order chromatin structure formed by histone acetylation and methylation marks. This result forms the basis for future investigation of the functional significance of these structural characteristics and how they are altered during different disease states.

## EXPERIMENTAL PROCEDURES

### Sample Preparation

MCF-10A cells were maintained in DMEM/F12 medium supplemented with 5% horse serum, 10 mg/mL insulin, 20 ng/mL EGF, 0.5 mg/mL hydrocortisone, and 100 ng/mL cholera toxin. MEFs (mouse embryonic fibroblasts) and U2OS (human bone osteosarcoma epithelial) cells were cultured in DMEM supplemented with 10% fetal bovine serum (FBS). Cell line was authenticated by STR DNA Profiling in Genetica DNA Laboratories. Gene loci profiles were verified using DSMZ reference databases. We first coated the glass-bottom dish (World Precision Instruments, FD3510) with 200 μL diluted 100-nm gold nanoparticle solution (1:60 with double-distilled water [ddH_2_O], EM.GC100, BBI Solutions) for 3 hr as the fiducial markers during STORM imaging (Ma et al., 2017). Then, cells were plated onto the dish at an initial confluency of about 50% and cultured overnight to let the cells attach to the dish.

To perform immunostaining, the culture medium was aspirated, and the cells were washed with PBS once and fixed in a 1:1 ethanol:methanol solution for 6 min at −20°C. After being washed once with PBS, the cells were blocked by incubation with blocking buffer (3% BSA, 0.05% Triton X-100 in PBS) for 2 hr and were then incubated with one or both primary antibodies diluted in blocking buffer at 4°C overnight. The cells were washed 3 times with washing buffer (0.2% BSA, 0.05% Triton X-100 in PBS) for 5 min per wash, and the corresponding secondary antibodies in the blocking buffer were added to the sample and incubated for 2 hr, protected from light. The cells were washed again three times with washing buffer, washed once with PBS for 5 min, and stored in PBS before imaging. Immediately before imaging, the buffer was switched to the STORM imaging buffer containing 10% (w/v) glucose (Sigma-Aldrich), 0.56 mg/mL glucose oxidase (Sigma-Aldrich), and 0.17 mg/mL catalase (Sigma-Aldrich). For single-color imaging, 0.14 M β-mercaptoethanol (Sigma-Aldrich) was used; for two-color imaging, 0.1 M mercaptoethylamine (MEA) (Sigma-Aldrich) was used. To exclude the potential fixation artifacts, the three most commonly used fixation methods for nuclear staining were evaluated. The STORM images of an active mark, a repressive mark, and active RNAP II from three fixation methods show similar structures of nanoclusters (see also Supplemental Experimental Procedures and Figure S6).

### Fluorescence Staining of DNA

DNA was stained by using the Click-iT Plus EdU (5-ethynyl-2′-deoxyuridine) Alexa Fluor 647 Imaging Kit (Thermo Fisher Scientific). The cells were plated onto a glass-bottom dish at an initial confluency of about 50% and cultured overnight to let the cells attach to the dish. Diluted Click-iT EdU reaction buffer in culture medium was added to the dish at a final concentration of 1 μM, and cells were incubated with EdU for 24 hr. After incubation, the media were removed and fixed with 4% paraformaldehyde for 15 min. Cells were washed 3 times with PBS and permeabilized with 0.2% Triton X-100 for 15 min. After washing the cells 3 times with 3% BSA in PBS, Click-iT Plus reaction cocktail was added to detect EdU. The cells were incubated with reaction cocktail for 30 min at room temperature and protected from light. The reaction cocktail was then removed and washed twice with 3% BSA in PBS. For two-color co-staining of DNA and histone marks, after being washed out of the reaction cocktail, cells were incubated with the primary antibody against the histone mark at 4°C overnight. The cells were then washed 3 times with the washing buffer (as described earlier in the Sample Preparation section) for 5 min per wash, and the corresponding Cy3B-conjugated secondary antibodies were added to the sample in blocking buffer and incubated for 2 hr at room temperature, being protected from light. The cells were washed again 3 times with washing buffer and once with PBS for 5 min per wash and stored in PBS before imaging.

Click-iT Plus reaction cocktails were prepared per the manufacturer’s instructions as follows: for a total volume of 500 μL, cocktails contain 440 μL 1X Click-iT reaction buffer, 10 μL copper protectant, 1.2 μL Alexa Fluor 647 picolyl azide, and 50 μL reaction buffer additive. All components were provided by the manufacturer’s imaging kits.

### Immunofluorescence Staining

All histone proteins were labeled using immunofluorescent staining method. The detailed protocols for immunofluorescent staining have been described in our previous publication (Xu et al., 2017). Alexa Fluor 647 conjugated to the secondary antibodies was used for single-color STORM imaging. For two-color STORM imaging of DNA and histone proteins, DNA was labeled by EdU and detected by Alexa Fluor 647 azide, and the detailed protocol was described in our previous publication (Ma et al., 2017). Histone proteins were immuno-stained by the appropriate primary antibodies and Cy3B-conjugated secondary antibodies, and the detailed protocol was previously described (Xu et al., 2017). For two-color STORM imaging based on dye pairs, secondary antibodies labeled with activator-reporter dye pairs (Alexa Fluor 405-Alexa Fluor 647 and Cy2-Alexa Fluor 647) were used, which were conjugated in our laboratory as previously described (Xu et al., 2017).

### Data Acquisition

Single-color and two-color STORM images were acquired using our custom-built system on an Olympus IX71 inverted microscope frame with a 100x, NA-1.4 oil immersion objective (UPLSAPO 100XO; Olympus), with each pixel on the camera corresponding to 130 nm on the sample plane, and fiducial markers were used for 3D drift correction as previously described (Ma et al., 2017). Prior to STORM imaging, the focal plane was adjusted so that the cells exhibit clearest nuclear periphery. For single-color STORM imaging, 40,000 frames were acquired at an exposure time of 20 ms. Two-color imaging was conducted sequentially; 30,000 frames of the Alexa Fluor 647 were acquired at an exposure time of 20 ms for each frame and followed with 30,000 frames of Cy3B at the same exposure time. The reconstruction of the super-resolution image was performed using our custom-written program written in MATLAB 2015 (MathWorks) and described in detail in our previous publication (Ma et al., 2017). The final reconstructed super-resolution image was rendered by accumulating all the valid molecules with a pixel size of 10 nm followed by a Gaussian smoothing filter (σ = 10 nm). To correct the chromatic aberration error across different color channels, a low density of multi-color fluorescence beads (TetraSpeck microspheres, 0.1-μm diameter, blue/green/orange/dark red fluorescence, Fisher Scientific) was used to generate the transform map in this paper (Sigal et al., 2015). The optical resolution of our system was characterized to be ~32 nm (see Figure S7).

Two-color STORM imaging using dye pairs was acquired with N-STORM (Nikon Instruments), as previously described (Xu et al., 2017). The samples were periodically activated with a sequence of 405-nm, 488-nm laser pulses and then imaged with a 647-nm laser. In each switching cycle, one of the activation lasers was turned on for 1 frame, followed by 3 frames of illumination with the red imaging laser. A total of 40,000 frames, including 10,000 activation frames and 30,000 imaging frames, for each channel were acquired. Imaging frames immediately after an activation pulse were recognized as a controlled activation event, and colors were assigned accordingly. A cross-talk subtraction algorithm was used to subtract the non-specific activation signal (Bates et al., 2007). The STORM imaging thickness is about 300 nm.

### Statistical Methods

The mean and SEM were calculated using Microsoft Excel. The statistical comparison between two groups was calculated using the Mann-Whitney U test in GraphPad Prism 7.0, and a two-tailed p value at 95% confidence interval was presented throughout the paper. A p value of less than 0.01 is considered significant.

### Calculation of RDF

The calculation of RDF, also known as pair-correlation function, was performed based on an established method written in MATLAB 2015 (MathWorks) (Caetano et al., 2015). In brief, for each cell nucleus, we divided the entire segmented cell nucleus into a maximum possible number of non-overlapping sub-regions with a size of 500 nm × 500 nm. Within each sub-region, we calculated the RDF by adapting the function “spatialStats” in the published software package MIiSR (Caetano et al., 2015). The RDF quantifies the density of localized spots as a function of distance (r) to other localized spots (self-clustering) based on the original coordinates of localized spots (or single molecules) from a single color. It illustrates the presence of multiple cluster sizes and intercluster distance without any assumptions on the shape of the clusters, and the relative degree of clustering is indicated by the height of the peaks corresponding to the molecular clusters (Caetano et al., 2015).

### Calculation of Mean Size and SD based on Gaussian Clustering

The details of the Gaussian clustering method have been described in our previous publication (Ma et al., 2017). Figure S7 shows the process of cluster analysis together with the selected parameters. For each cell nucleus, the histogram of the cluster size for each of the histone marks was plotted and fitted with a log-normal distribution (Figure S8). The mean size and SD were calculated by averaging the values from ~20 to 40 nuclei for each histone mark. The histogram ofthe nearest neighbor distances (nnds) ofeach histone mark was shown in Figure S8.

### Calculation of Co-localization

We calculated the DoC (shown in Figures 4 and 5) between the histone marks and active RNAP II, based on a published algorithm (Malkusch et al., 2012; Pageon et al., 2016), written in MATLAB 2015 (MathWorks). The original coordinates of localized spots (or single molecules) from two colors were used as the basis for our calculation. In brief, for each localized spot from a histone mark, we first calculated the gradient density of the histone mark and RNAP II around this localized spot, based on the number of localized spots from the histone mark and RNAP II within circles of increasing radius (a range of 20 to 500 nm at a step size of 10 nm was used), respectively. This gradient density of the histone mark and RNAP II was normalized by their respective gradient density within the area with the maximum radius and then used to calculate the Spearman correlation. The DoC score ranging from –1 (anti-correlated) to 1 (correlated) with respect to the histone mark was assigned to each localized spot.

## Supplementary Material

1

## Highlights

- Histone marks form three distinct types of structures in interphase and during mitosis
- Active histone marks coincide with less compact DNA and near active RNA polymerase II
- Repressive marks coincide with condensed DNA and distant from active RNA polymerase II
