# Supplementary material for: Super-Resolution Imaging of Higher-Order Chromatin Structures at Different Epigenomic States in Single Mammalian Cells
Source: Cell Rep. Author manuscript; Available in PMC 2018 Sep 25. (PMC6154382; doi:10.1016/j.celrep.2018.06.085)
Supplement: 1 [file NIHMS1503262-supplement-1.pdf]

Cell Reports, Volume *24*

Supplemental Information

Super-Resolution Imaging of Higher-Order Chromatin Structures at Different Epigenomic States in Single Mammalian Cells

Jianquan Xu, Hongqiang Ma, Jingyi Jin, Shikhar Uttam, Rao Fu, Yi Huang, and Yang Liu

SUPPLEMENTAL FIGURES

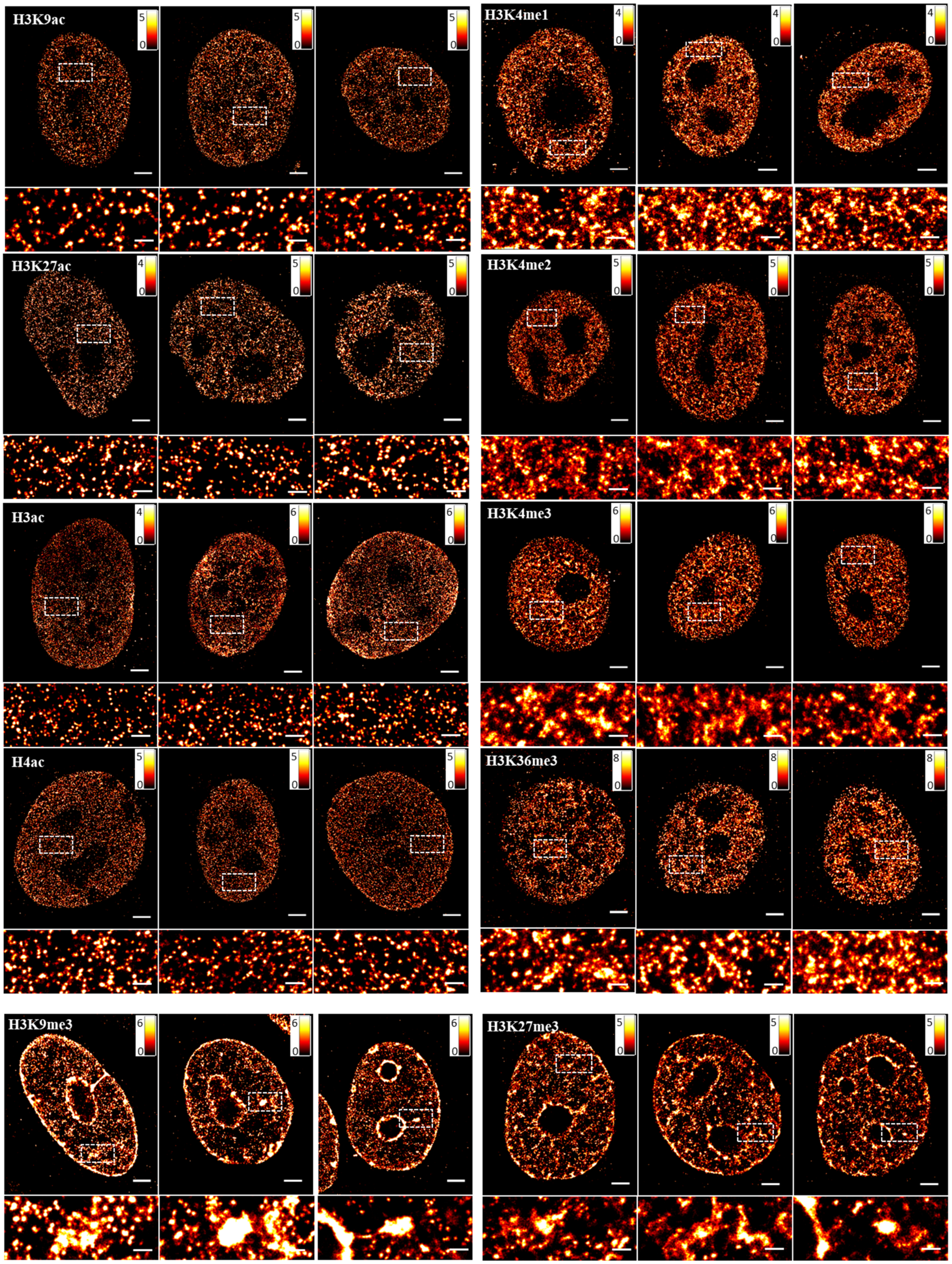

Figure S1. Additional STORM images of different histone marks. Related to Figure 1. Scale bar is 2 µm and 500 nm in the original and magnified images, respectively.

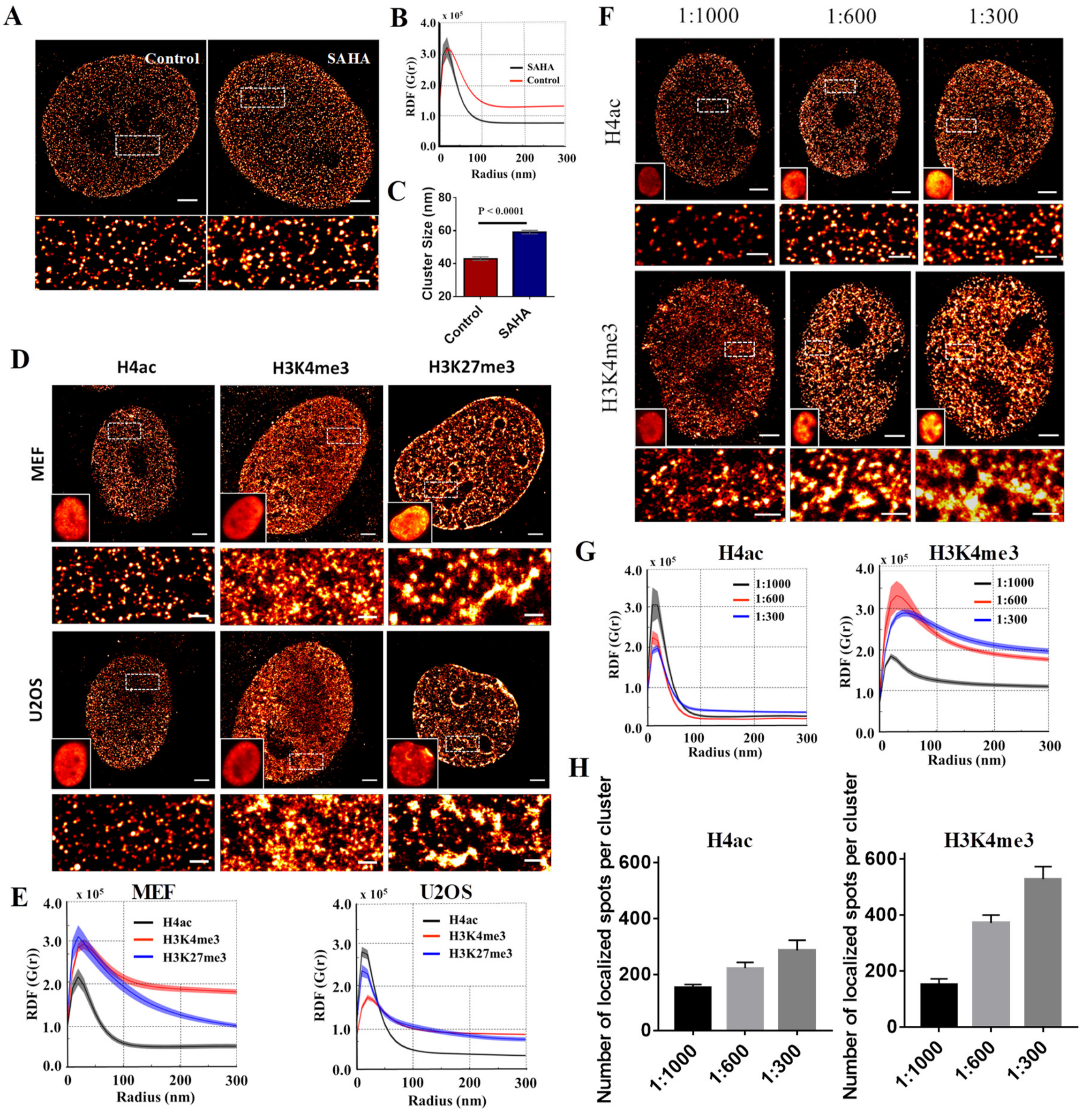

Figure S2. Validation of chromatin structure at different epigenomic states by SAHA treatment, different cell lines, and label density. Related to Figure 1. (A) Representative STORM images of H4ac treated with or without suberoylanilide hydroxamic acid (SAHA). **(B)** Radial distribution function (RDF) of the H4ac clusters, shaded area showing the standard error. **(C)** Statistical analysis of the cluster size of H4ac with or without SAHA treatment (~30 cells). Data are represented as mean ± standard error, and the *P* values were determined using Mann-Whitney test. **(D)** Representative STORM images of H4ac, H3K4me3 and H3K27me3 stained on different cell lines (mouse embryonic fibroblast (MEF) and U2OS cells). **(E)** The RDF for the three histone marks of MEF and U2OS cells. **(F)** Representative STORM images of H4ac and H3K4me3 stained with varying label density (primary antibody dilution: 1:1000, 1:600, 1:300). **(G)** The RDF for the three different antibody concentrations. The distinction between H4ac and H3K4me3 is consistent for all antibody concentrations. **(H)** The number of localized spots per cluster as a function of antibody concentration. Scale bar is 2 µm and 500 nm in the original and magnified images, respectively.

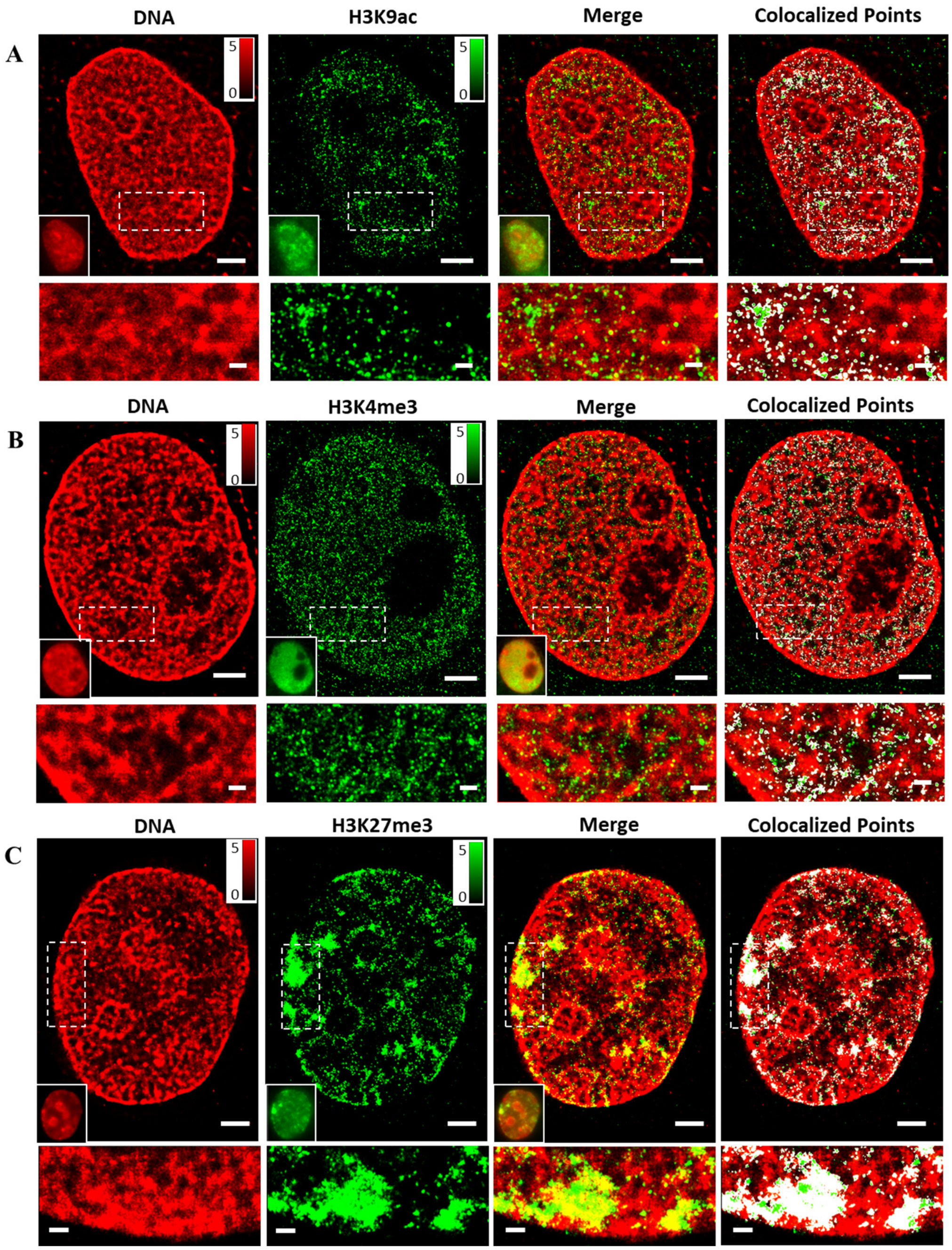

Figure S3. Additional two-color STORM images of DNA and different histone marks. Related to Figure 2. Representative STORM images showing spatial relationship between DNA compaction and different histone marks - (A) H3K9ac, (B) H3K4me3, (C) H3K27me3. White spots show the colocalized point. Scale bar is 2 μm and 500 nm in the original and magnified images, respectively.

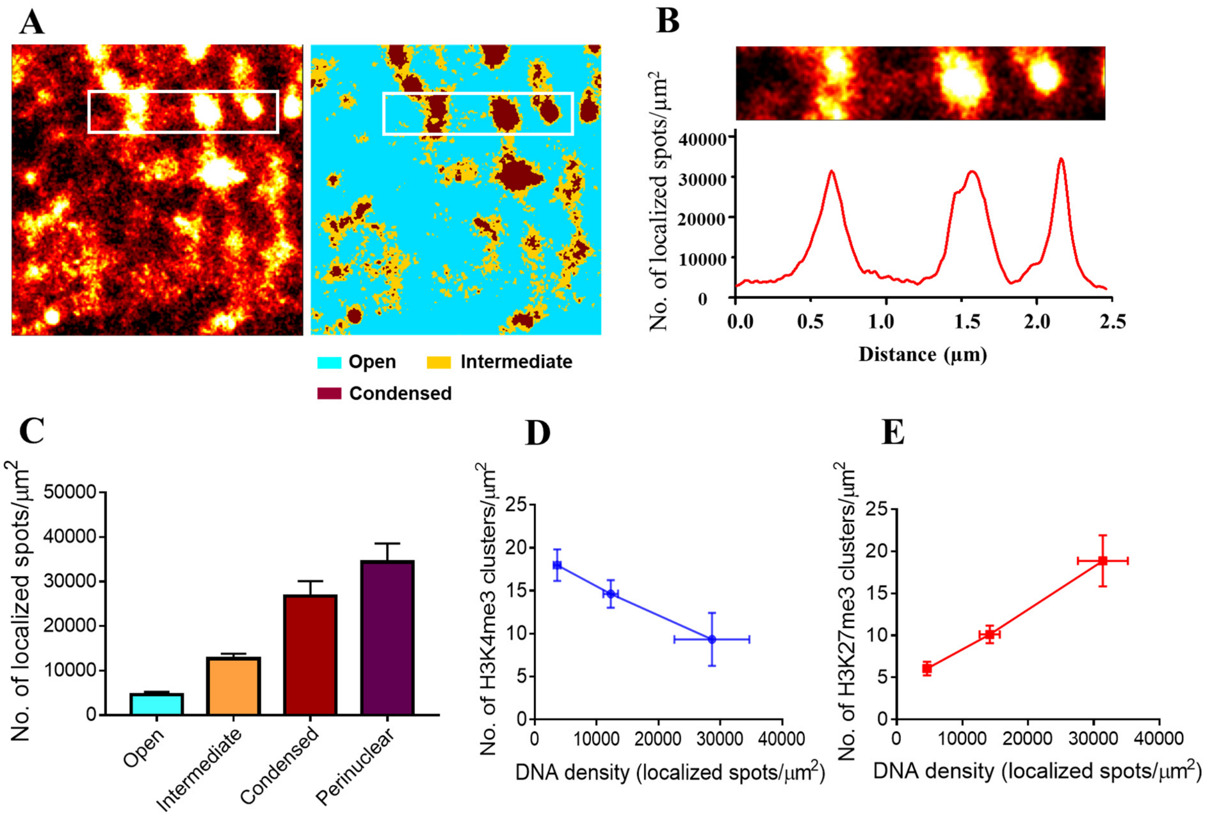

Figure S4. Quantitative analysis of chromatin structure and its relationship to active and repressive histone marks. Related to Figure 2. (A) The super-resolution image of DNA and the corresponding three compaction levels (open (cyan), intermediate (brown) and condensed (dark red) chromatin) classified based on k-means clustering (using Matlab "kmeans" function. **(B)** Cross-sectional profile of the DNA density (number of localized spots/μm^2) for the region shown in the box. **(C)** The average DNA density (number of localized spots/μm^2) in open, intermediate, condensed chromatin and the perinuclear region (nuclear periphery). The error bar is standard error. **(D-E)** Cluster density (number of clusters/μm^2) of active histone mark (H3K4me3) and repressive histone mark (H3K27me3) as a function of DNA density. The error bar is standard error. The number of H3K4me3 and H3K27me3 clusters was calculated within each classified open, intermediate and condensed chromatin region. The clustering method is described in Methods. The cluster density of the active histone mark shows a progressive decrease over the increased DNA density; while the cluster density of the repressive histone mark shows the opposite trend.

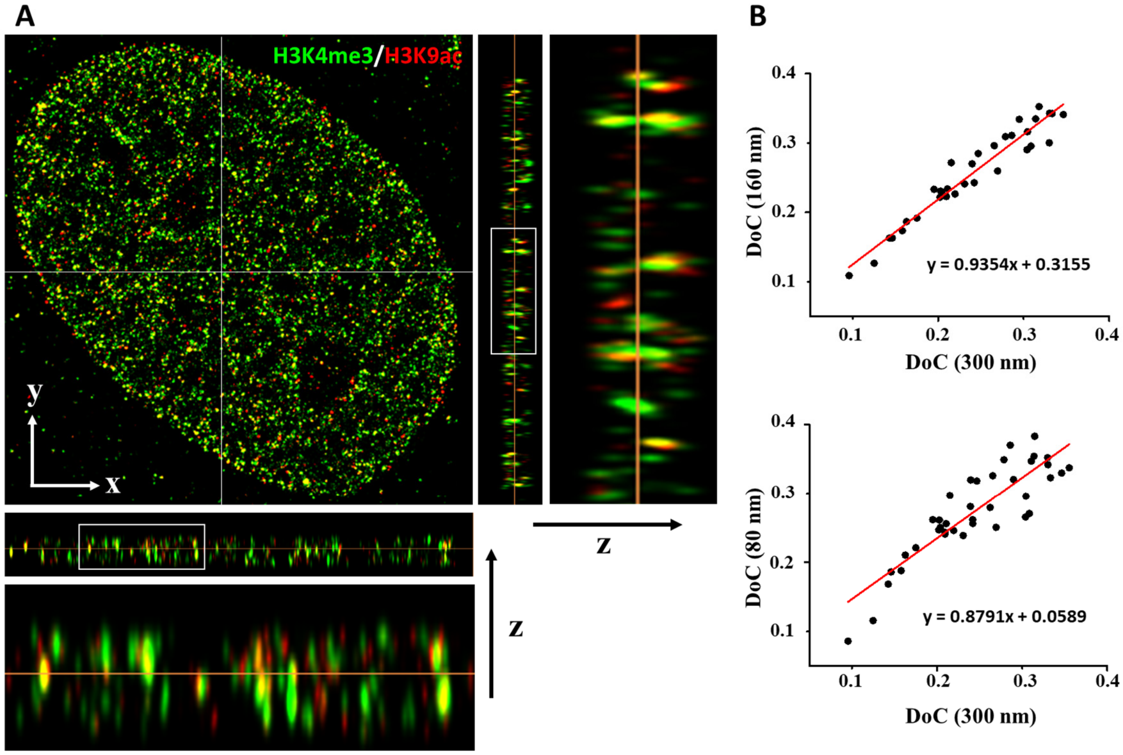

Figure S5. Validation of projection effect on the measured co-localization from the 2D images by astigmatism-based two-color 3D-STORM imaging. Related to Figure 4 and Figure 5. (A) Representative two-color cross-sectional (xy-plane, xz-plane and yz-plane) STORM images of two active histone marks (H3K4me3 and H3K9ac) obtained from 3D-STORM imaging, and the zoomed images along the axial (z) direction. It can be seen that most histone marks co-localized in the xy-plane is also co-localized along the axial direction. **(B)** Comparison of degree of co-localization calculated from the 2D STORM images projected from 300 nm, with that calculated from 160 nm and 80 nm thick sections.

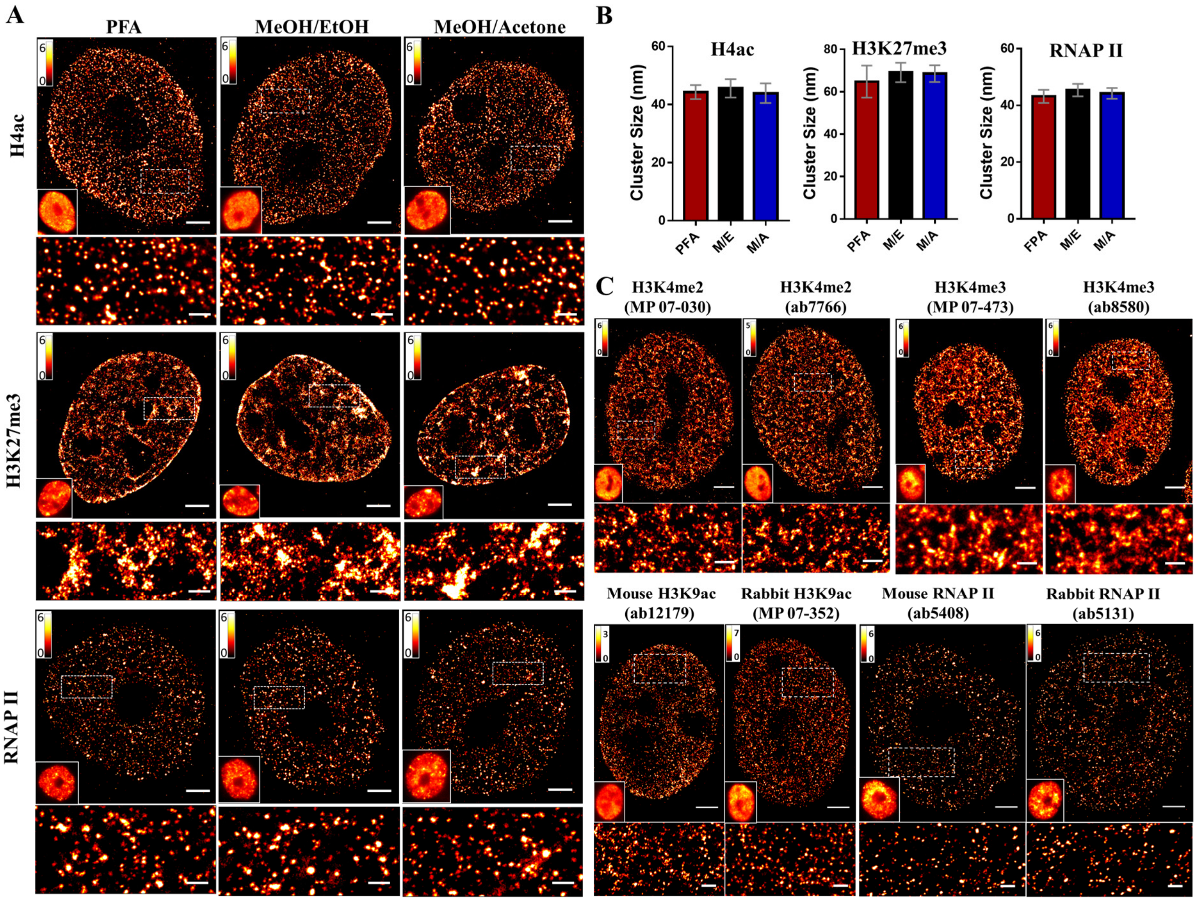

Figure S6. Validation of fixation methods and antibodies. Related to Experimental Procedures. (A) Representative STORM images of H4ac, H3K27me3 and active RNAP II, fixed with three different methods, including 4% paraformaldehyde (PFA), Methanol/Ethanol (MeOH/EtOH) and Methanol/Acetone (MeOH/Acetone). **(B)** Statistical analysis of cluster size of different fixation methods (7 cells analyzed). Error bars indicate standard error. **(C)** Representative STORM images showing the same proteins labeled with antibodies from different manufacturers and different host specimens (mouse or rabbit). scale bar: 2 µm and 500 nm in the original and magnified images, respectively.

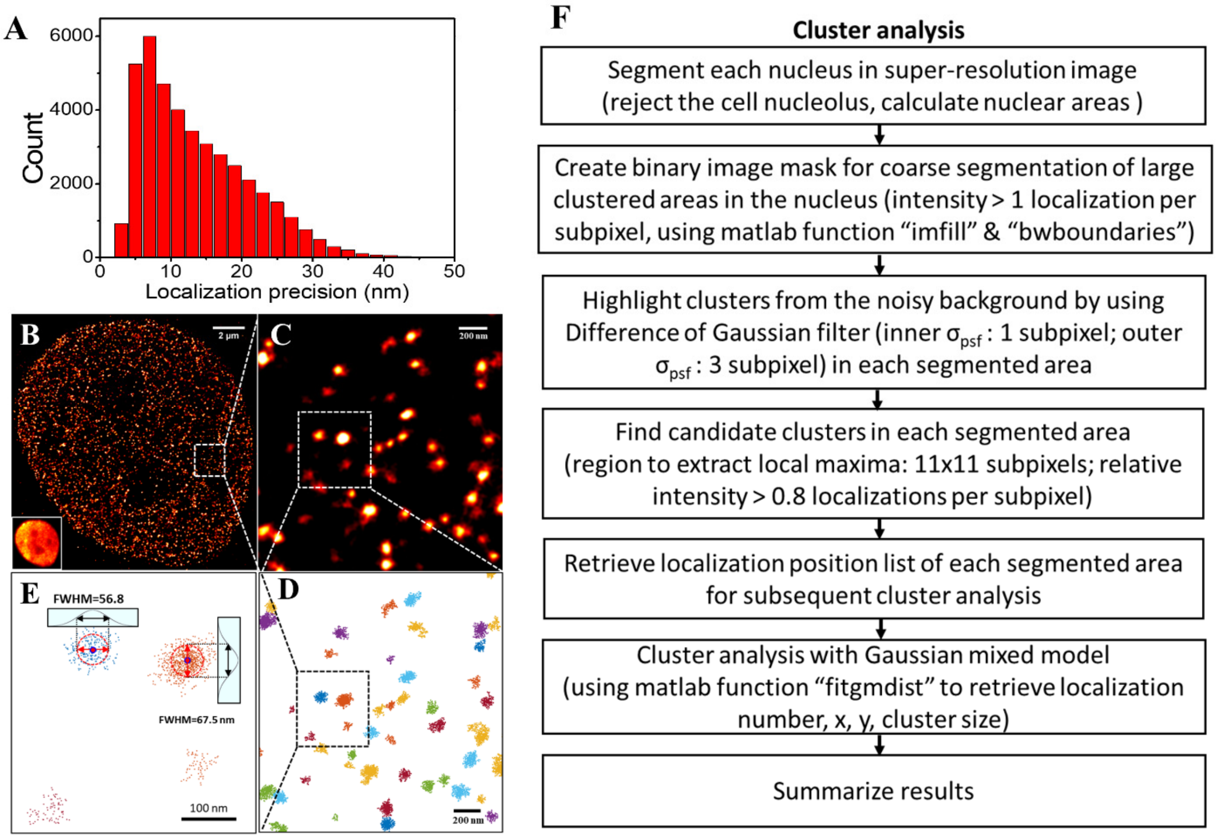

Figure S7. Imaging resolution, calculation of cluster size and procedure of cluster analysis. Related to Figure 1, and Experimental Procedures. (A) Histogram of localization precision (mean value = 13.5 nm) for STORM imaging of histone marks, corresponding to an optical resolution of ~32 nm. **(B-E)** Cluster analysis to automatically identify and quantity the structures marked by histone marks. **(B)** The reconstructed super-resolution image of H4ac. **(C)** The magnified region of the white box in **(B)**. **(D)** The identified clusters marked by different colors from the same region in **(C)**, and each dot represents each localized spot. **(E)** Estimation of the equivalent cluster size defined by the full-width at half maximum (FWHM) of each cluster from the magnified region of the black box in **(D)**. **(F)** Flowchart to detail the steps for cluster analysis.

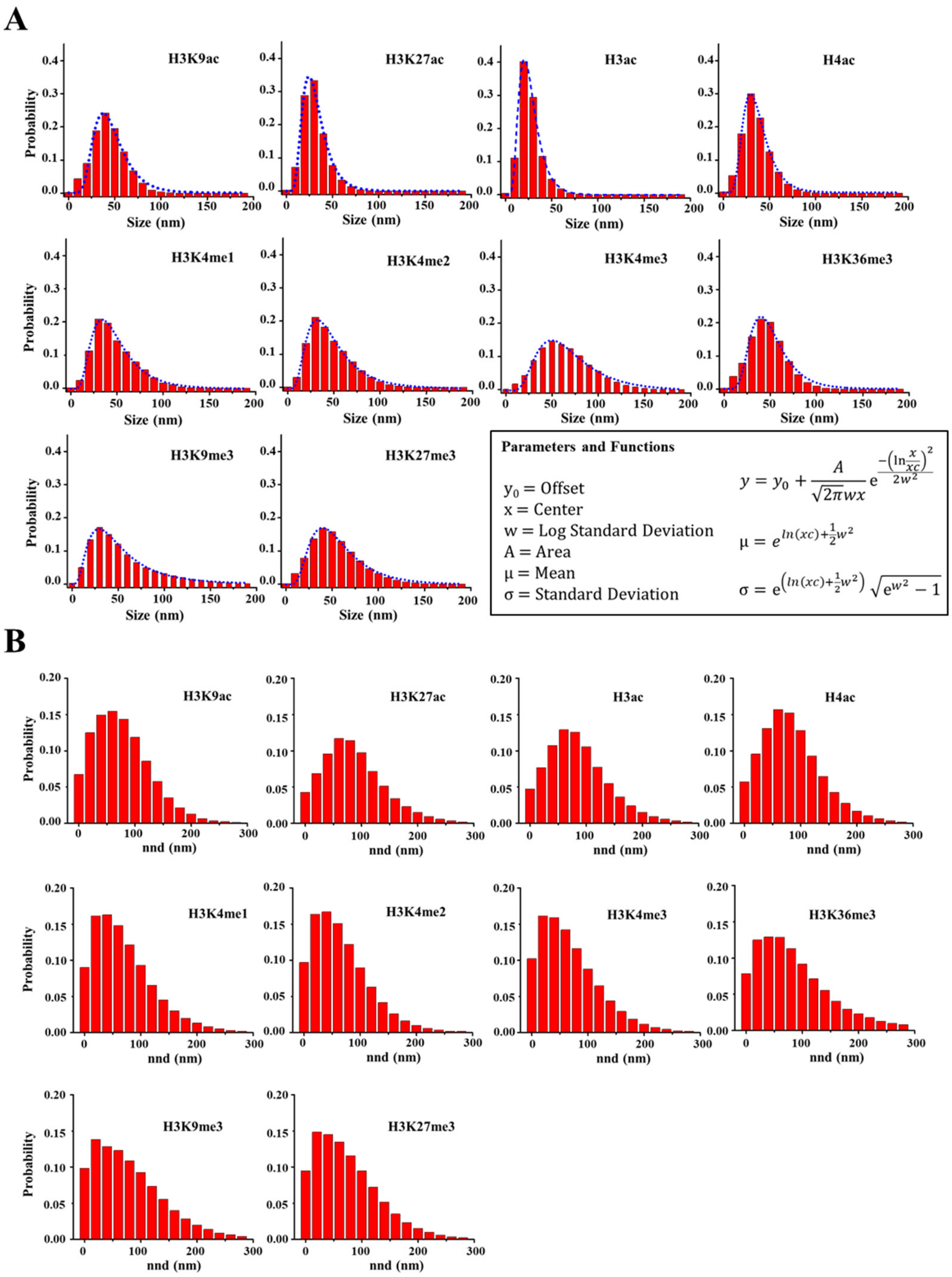

Figure S8. Histogram distribution of cluster size and nearest neighbor distance (nnd) of different histone marks. Related to Figure 1. (A) The mean histogram distribution of cluster size of 10 histone marks fitted with a lognormal function. The figure shows the parameters and functions used to calculate the mean size and standard deviation, which were used in Fig. 1E of the main text. **(B)** The mean histogram distribution of nearest neighbor distances (nnd) for 10 histone marks. The nearest neighbor distance is defined as the distance between the center of two closest clusters subtracting their respective cluster size.

Table S1. List of primary antibodies used in this study. Related to Experimental Procedures.

Target	Host	Company	Catalog number	Dilution used	Images in Figures
H3K27me3	Rabbit	EMD Millipore	07-449	1:600	Fig. 1, Fig. 4, Fig 5. Fig. S1, Fig. S2, Fig S6.
H3K27me3	Rabbit	Cell Signaling	9733	1:600	Fig. 2, Fig. 3, Fig. S3, Fig. S6.
H3K4me1	Rabbit	abcam	ab8895	1:600	Fig. 1, Fig. S1
H3K4me3	Rabbit	EMD Millipore	07-473	1:600	Fig. 1, Fig. 2, Fig3. Fig 4 (H3K4me3/H3K9ac), Fig. 5. Fig. S1, Fig. S2, Fig. S3, Fig. S5, Fig. S6.
H3K4me3	Rabbit	abcam	ab8580	1:600	Fig. S2
H3K4me3	Mouse	EMD Millipore	05-1339	1:200	Fig. 4 (H3K27me3/H3K4me3)
H3K4me2	Rabbit	abcam	ab7766	1:600	Fig. S2
H3K4me2	Rabbit	EMD Millipore	07-030	1:600	Fig. 1, Fig. S1. Fig. S2
H4ac	Rabbit	EMD Millipore	06-598	1:600	Fig. 1, Fig. 3, Fig. 5, Fig. S1, Fig. S2, Fig. S2, Fig. S6, Fig. S7.
H3ac	Rabbit	EMD Millipore	06-599	1:600	Fig. 1, Fig. S1.
H3K27ac	Rabbit	abcam	ab177178	1:600	Fig. 1, Fig. S1.
H3K9me3	Rabbit	abcam	ab8898	1:600	Fig. 1, Fig. 3, Fig. 5. Fig. S1.
H3K9ac	Rabbit	EMD Millipore	07-352	1:600	Fig. 1, Fig. 2, Fig. 3, Fig S1. Fig. S3, Fig. S6.
H3K9ac	Mouse	abcam	ab12179	1:200	Fig. 4, Fig. 5, Fig. S5. Fig. S6.
H3K36me3	Rabbit	abcam	ab9050	1:300	Fig. 1, Fig. 5, Fig. S1.
RNAP II (phospho S5)	Mouse	abcam	ab5408	1:600	Fig. 5 (except H3K9ac/RNAP II), Fig. S6.
RNAP II (phospho S5)	Rabbit	abcam	ab5131	1:600	Fig. 5 (H3K9ac/RNAP II), Fig. S6.

SUPPLEMENTAL EXPERIMENTAL PROCEDURES

Validation of antibodies

For STORM imaging, we chose highly-purified or ChIP-Grade antibodies with validated specificity and efficiency from different companies. For two-color STORM imaging, as antibodies from different hosts are required, we validated that the same histone mark labeled by antibodies from different hosts exhibit comparable structural characteristics (shown in Figure S6). All primary antibodies used in this study are listed in Table S1.

Validation of fixation methods

To confirm that the super-resolution images was not affected by different sample preparation approaches, we performed three most common used fixation methods in nuclear staining including paraformaldehyde (PFA), Methanol/Acetone (v/v 1:1), and Methanol/Ethanol (v/v 1:1). By taking H4ac, H3K27me3 and RNAP II as illustrative examples, we show that the cluster structure is independent of the fixation and permeabilization protocols used. As shown in Figure S6 the STORM images from three fixation methods show similar structures of nanoclusters with similar size.

Treatment with histone deacetylase inhibitor

The cells were plated onto a glass-bottom dish at an initial confluency of about 50%. After overnight incubation, SAHA (Suberoylanilide Hydroxamic Acid. Cayman Chemical) was added to achieve final concentrations of 1 μM. Control cells were maintained with an equal volume of full culture medium. After 24 hours, cells were fixed and immunostaining was then performed as described above.

Segmentation of cell nuclei

Each nucleus in the reconstructed STORM images was segmented via an automatic segmentation algorithm written in MATLAB 2015 (MathWorks Inc). Our algorithm exploits the idea of aggregation by performing elliptical parameterization of the nucleus through the Eigen decompostion of the coordinates of the chromatin clusters. The resulting estimates of the major and minor axes is transformed to polar coordinates to compute the boundary of the nucleus. We empirically found that this eigen-based aggregation approach was able to correctly identify the majority of the chromatin clusters within the nucleus, except those clusters right on or near the boundary, where background staining becomes a confounding factor. To consistently exclude background staining, we applied a center-of-mass based local criterion on our initial boundary estimate, allowing us to robustly estimate it in a single boundary traversal. Specifically, we started with the initial boundary estimate and performed dilation and erosion on it to respectively get larger and smaller nuclei. The region of the nucleus between the two defined an annulus with the initial boundary estimate defining the principal curve of the chromatin clusters that lay in this annulus. We performed a single traversal on the principal curve, computing at each of its points the local estimate of the center-of-mass of the clusters. If the center-of-mass lay beyond the corresponding point on the principal curve, the nucleus boundary was locally expanded to the outer ring of the annulus, and if the opposite was true it was shrunk to inner ring. The expansion and shrinkage were performed smoothly using spline interpolation. The resulting boundary defined the segmented nucleus. An additional layer of robustness was provided through a graphical user interface for the expert to quickly visualize the segmentation results and adjust them, if necessary. We found that on most occasions the segmentation was accurate. In cases where the automatic segmentation algorithm does not perform, we manually segmented the cell nuclei.
